# Antimicrobial resistance in coagulase-negative staphylococci from Nigerian traditional fermented foods

**DOI:** 10.1186/s12941-017-0181-5

**Published:** 2017-01-31

**Authors:** P. T. Fowoyo, S. T. Ogunbanwo

**Affiliations:** 1 Biosciences Department, Salem University, P.M.B. 1060 Lokoja, Kogi State Nigeria; 2 Microbiology Department, University of Ibadan, Ibadan, Oyo State Nigeria

**Keywords:** Antibiotic resistance, Coagulase-negative staphylococci, Fermented foods, Methicillin resistance, *mecA* gene

## Abstract

**Background:**

Coagulase-negative staphylococci have become increasingly recognized as the etiological agent of some infections. A significant characteristic of coagulase-negative staphylococci especially strains isolated from animals and clinical samples is their resistance to routinely used antibiotics although, resistant strains isolated from fermented foods have not been fully reported.

**Methods:**

A total of two hundred and fifty-five CoNS isolates were subjected to antimicrobial susceptibility test using the disc diffusion technique. The minimum inhibitory concentration of the isolates to the tested antibiotics was determined using the microbroth dilution method. Methicillin resistant strains were confirmed by detection of methicillin resistant genes (*mecA*) and also employing cefoxitin screening test.

**Results:**

The isolates were confirmed to be methicillin resistant by the detection of *mecA* genes and the cefoxitin screening test. The isolates demonstrated appreciable resistance to ampicillin (86.7%), sulfomethoxazole–trimethoprim (74.9%), amoxicillin–clavulanic acid (52.5%) and oxacillin (35.7%). Methicillin resistance was exhibited by 13 out of the 255 isolates although no *mecA* gene was detected. It was also observed that the methicillin resistant isolates were prevalent in these traditional foods; *iru*, *kindirmo*, *nono* and *wara*.

**Conclusion:**

This study has ameliorated the incidence of multiple antibiotic resistant coagulase-negative staphylococci in Nigerian fermented foods and if not tackled adequately might lead to horizontal transfer of antibiotic resistance from food to man.

## Background

Coagulase-negative staphylococci were previously dismissed as contaminants and were found to occur mostly in hospitalized patients, individuals suffering from nosocomial infections and infections arising from the use of catheter or other intra-uterine devices however, it has been shown that CoNS from fermented foods also exhibit virulent traits [1]. The major challenge of CoNS-related infections has been the difficulty in therapy due to antimicrobial resistance. Antimicrobial agents used in therapy and as feed supplements to promote growth in food animals may increase the spread of drug-resistant bacteria. Such bacteria may contaminate milk or meat and are subsequently found in fermented food made of such raw material [2]. The levels of antibiotic resistant infections in the developing world have increased steadily in the last few decades as a result of combination of microbial characteristics and the selective pressure of antimicrobial use [3]. Microbial mechanisms of overcoming the activities of antimicrobial agents include the production of structure-altering or inactivating enzymes (e.g. beta-lactamase or amino glycoside-modifying enzymes), alteration of penicillin-binding proteins or other cell-wall target sites, altered DNA gyrase targets, permeability mutations, active efflux and ribosomal modification [4–6]. Multidrug-resistant bacteria in both the hospital and community environment are important concern to the clinician, as it is the major cause of failure in the treatment of infectious diseases, increased morbidity, and mortality and the evolution of new pathogens [7, 8].

Penicillin was initially the drug of choice for treatment of infections caused by *Staphylococcus* however, penicillin resistance in CoNS became very high since 1968 [9]. Nowadays, resistance is around 91% in clinical strains [10]. Two mechanisms confer penicillin resistance in staphylococci; the first and the most important is the production of β-lactamase which inactivates penicillin by the hydrolysis of its β-lactam ring. The second is primarily associated with human isolates and confers resistance due to a penicillin-binding protein, PBP2a, encoded by *mecA* [2]. The *blaZ* has also been identified as the cause of penicillin resistance among coagulase-negative staphylococci (CoNS) suggesting that *blaZ* is one of the main mechanism of penicillin resistance in staphylococci [2]. Methicillin resistance in *Staphylococcus* is caused by the expression of PBP2a encoded by the *mecA* gene [11]. Resistance of staphylococci to methicillin and all β-lactam antibiotics is associated with the low affinity of a penicillin-binding protein, PBP2a, which is not present in susceptible staphylococci [12–17]. This protein is encoded by the *mecA* gene, which is located in the mec region in which the DNA is of foreign origin [18]. There is evidence of horizontal transfer of SCC cassette between staphylococcal species [19] which implies that CoNS could serve as a reservoir for the spread of resistance genes. Transfer of resistance genes between CoNS and *S. aureus* has been reported thus indicating that CoNS may act as a resistance gene reservoir for *S. aureus*. It is thus possible that the different species of staphylococci that are present in the same microenvironment, for example on the skin of dairy cows can exchange *mecA* and *blaZ*, if the appropriate bacterial factors are met [2].

In this study, the incidence of antibiotic resistance against 9 antibiotics among 255 strains of coagulase-negative staphylococci of fermented food associated CoNS were investigated using disc diffusion technique according to the CLSI guidelines. The antibiotic resistant phenotypes were confirmed molecularly by the detection of *mecA* genes and cefoxitin screening test.

## Methods

### CoNS strains used in this study

In total, 255 CoNS strains were used in this study. The strains were isolated from six different Nigerian fermented foods, including *kindirmo* (66), *nono* (44) *iru* (58), *wara* (32), *ogi* (28) and *kunu* (27). The isolates were identified using both conventional and molecular methods employing 16S rRNA sequencing [20].

### Antibiotic susceptibility testing

*In vitro* susceptibility of the test isolates to the antibiotics was determined using Kirby-Bauer disc diffusion [21]. A sterile wire loop was used to pick a discrete colony of the 18 h old culture of each of the test isolate cultured on mannitol salt agar (MSA) and used to inoculate sterile brain heart infusion broth inside a test tube and incubated at 37 °C for 4 h. The inoculum was standardized using the 0.5 McFarland turbidity standard which corresponds to 1.5 × 10^8^ cfu/ml of cells. A sterile cotton swab was dipped into the adjusted suspension and excess inoculum was removed by pressing the swab firmly on the inside wall of the tube. The dried surface of a Mueller–Hinton agar plate was inoculated by streaking the swab over the entire surface. This procedure was repeated by streaking two more times, rotating the plate approximately 60º each time to ensure an even distribution of inoculum. The antimicrobial discs were placed firmly on the surface of the inoculated agar plate using sterile forceps. The plates were left for 1 h after which they were incubated at 35 °C for 18 h. After 16–18 h of incubation, the plates were examined and the diameters of the zones of inhibition were measured. The discs used were ampicillin (30 µg), amoxicillin-clavulanic acid (30 µg), cefotaxime (30 µg), oxacillin (1 µg), ciprofloxacin (5 µg), trimethoprim–sulphomethaxazole (5 µg), erythromycin (25 µg), gentamycin (10 µg) and ofloxacin (5 µg). All the antibiotic discs were procured from Oxoid, Germany. The results were classified as susceptible, intermediate, or resistant according to the approved guidelines of the Clinical and Laboratory Standards Institute [22].

### Determination of minimum inhibitory concentration (MIC) using the broth micro-dilution method

The method by [21] was employed. A 96-well microtitre plate was used. Twofold serial dilutions of the different antibiotics were prepared and dispensed into the microtitre plates. The antimicrobial solutions were prepared at twice the desired final concentration and the wells filled with 0.05 ml of the antibiotic instead of 0.1 ml. Each tray labeled had a growth control well and a sterility (uninoculated) well.

The inoculum used for the broth micro-dilution was prepared using the direct colony suspension method. An 18 h old culture of CoNS was grown on blood agar. Distinct colonies were picked and each inoculated into 5 ml of Mueller–Hinton broth in a test tube. The broth culture was incubated at 35 °C for 4 h. The turbidity of the actively growing broth culture was adjusted with sterile broth using 0.5 McFarland standard. The resulting suspension contained approximately 1–2 × 10^8^ cfu/ml. 2 ml of the suspension was dispensed into 38 ml of water (1:20 dilution). The prong of the inoculator was used to transfer 0.01 ml (1:10 dilution) into each well. The MIC panel was inoculated carefully to avoid splashing from one well to another. The microdilution trays were incubated inside a plastic bag at 35 ± 2 °C for 16–20 h in an ambient air incubator within 15 min of adding the inoculum. The amount of growth in the wells containing the antimicrobial agent was compared with the amount of growth in the growth control wells (no antimicrobial agent) used in each set of tests when determining the growth end points. A test was considered valid when acceptable growth was ≥2 mm turbidity at the bottom of the well or when a definite turbidity was observed.

### Detection of methicillin resistance genes (*mecA*)

The methicillin resistant genes present in the coagulase-negative staphylococci strains were detected by polymerase chain reaction according to the method of [23]. DNA was extracted using the QIA Amp mini kit (QIAgen). Polymerase chain reaction for detection of the gene *mecA* (513 bp) was carried out using the following primers: A22f (5′ AAA ATC GAT GGT AAA GGT TGG C 3′) and A22r (AGT TCT GCA GTA CCG GAT TTG C) as described by [11]. Amplification cycles for *mecA* was carried out according to the method of [23] Considering 40 cycles of 94 °C for 30 s, 55 °C for 30 s, 72 °C for 1 min with a final extension of 72 °C for 5 min. *Staphylococcus aureus* ATCC43300 *mecA* + was used as positive control [21]. The amplicons were evaluated by agarose gel electrophoresis followed by staining in ethidium bromide (0.5 mg/ml), visualized on UV transilluminator (UVP, Inc USA) and documented by the program QuantiOne (BioRad) using molecular weight markers of 100 bp (Fermentas ^®^).

## Results

A total of 221 (86.7%) of the isolates were resistant to ampicillin, however majority of the resistant CoNS occurred in *wara* (93.8%), *nono* (88.6%), *kindirmo* (92.4%) and *iru* (86.2%). 74.9% of the CoNS isolates were resistant to trimethoprim-sulfamethoxazole with high incidence in *iru* (84.5%), *wara* (84.4%) and *kindirmo* (72.7%). The highest resistance of amoxicillin-clavulanic acid was noted in CoNS isolated from *ogi* (60.7%), *iru* (60.3%), *nono* and *wara* (59.4%). The highest oxacillin resistance isolates were from *ogi* (42.9%), *nono* (40.9%) and *wara* (43.8%). The resistance to the other antibiotics cefotaxime, ciprofloxacin, erythromycin, gentamycin and ofloxacin were not as high as the other antibiotics as shown in Table 1.

**Table 1:** Distribution and Percentage Antimicrobial Resistance of Coagulase-Negative Staphylococci from Fermented Food Samples

Antibiotics	% Resistance of CoNS from foods						
	Total	*Iru*	*Ogi*	*Nono*	*Kindirmo*	*Kunu zaki*	*Wara*
	(255)	n = 58	n = 28	n = 44	n = 66	n = 27	n = 32
Ampicillin	221 (86.7%)	50 (86.2%)	21 (75%)	39 (88.6%)	61 (92.4%)	20 (74.1%)	30 (93.8%)
Trimethoprim–sulfamethoxazole	191 (74.9%)	49 (84.5%)	18 (64.3%)	31 (70.5%)	48 (72.7%)	18 (66.7%)	27 (84.4%)
Amoxicillin–clavulanic acid	134 (52.5%)	35 (60.3%)	17 (60.7%)	26 (59.1%)	29 (43.9%)	8 (29.6%)	19 (59.4%)
Cefotaxime	9 (3.5%)	2 (3.4%)	0 (0%)	3 (6.8%)	2 (3.0%)	1 (3.7%)	1 (3.1%)
Oxacillin	91 (35.7%)	17 (29.3%)	12 (42.9%)	18 (40.9%)	21 (31.8%)	9 (33.3%)	14 (43.8%)
Ciprofloxacin	61 (23.9%)	12 (20.7%)	3 (10.7%)	10 (22.7%)	18 (27.3%)	5 (18.5%)	13 (40.6%)
Erythromycin	40 (15.7%)	8 (13.8%)	2 (7.1%)	10 (22.7%)	11 (16.7%)	0 (0%)	9 (28.1%)
Gentamicin	29 (11.4%)	4 (6.9%)	2 (7.1%)	9 (20.5%)	7 (16.7%)	0 (0%)	7 (21.9%)
Ofloxacin	18 (7.1%)	2 (3.4%)	1 (3.6%)	4 (9.1%)	4 (6.1%)	1 (3.7%)	6 (18.8%)

**Fig. 1:**
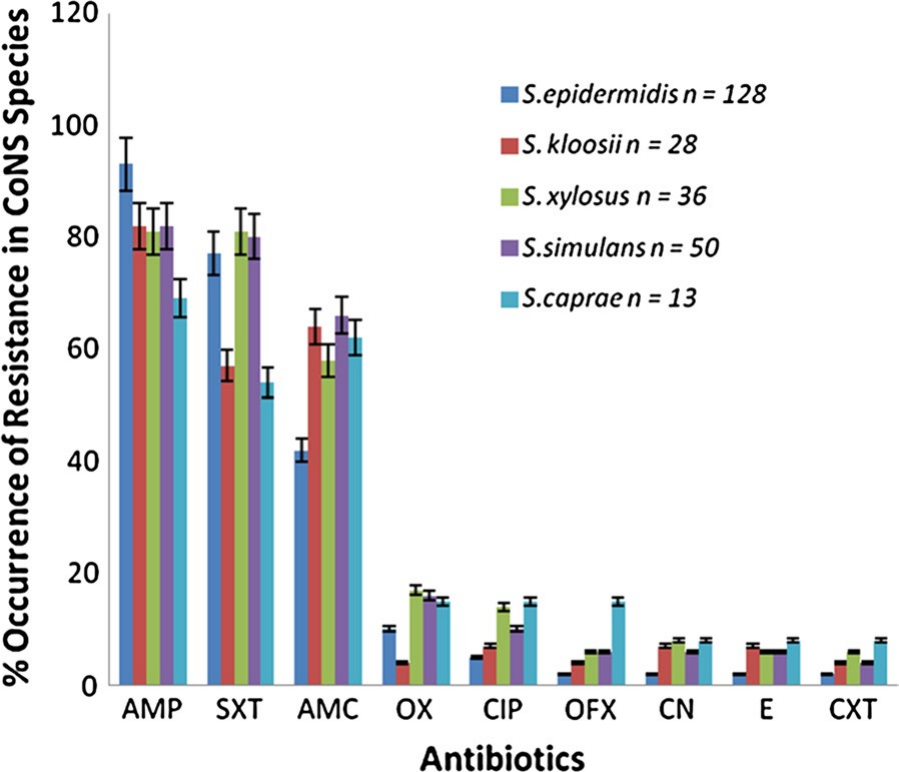
Percentage occurrence of antibiotic resistance in CoNS Species. *Error bars* represent standard deviation of mean of replicate determinations

Table 2 shows the resistance phenotype of the CoNS species. Thirty-four (13%) of the isolates were not resistant to any of the antibiotic tested. CoNS species having resistance phenotype to only ampicillin and trimethoprim-sulfamethoxazole were only 57 in number. *Staphylococcus epidermidis* (92%) demonstrated the highest resistance to ampicillin while *S. caprae* (69%) had the least percentage of resistance to ampicillin. For trimethoprim-sulfamethoxazole, the highest resistance of 81% was recorded in *S. xylosus* with *S. caprae* having the least resistance (53%) to the antibiotic. *Staphylococcus simulans* (68%) recorded the highest resistance to amoxicillin-clavulanic acid while the least resistance was shown in *S. epidermidis* (41%). Oxacillin resistance in *S. xylosus* was 32% which was the highest and the least was in *S. kloosii* (14%). *Staphylococcus caprae* exhibited the highest resistance to ciprofloxacin, ofloxacin, gentamycin, erythromycin and cefotaxime as shown in Fig. 1.

**Table 2:** Phenotypic antimicrobial resistance patterns of CoNS species from fermented food samples

Profile	Resistance phenotypes	Number of strains	Number of antibiotic classes
1	None	34	0
2	AMP	30	1
3	AMP, SXT	57	2
4	AMP, SXT, AMC	43	2
5	AMP, SXT, AMC, OX	30	2
6	AMP, SXT, AMC, OX, CIP	21	3
7	AMP, SXT, AMC, OX, CIP, E	11	4
8	AMP, SXT, AMC, OX, CIP, E, CN	10	5
9	AMP, SXT, AMC, OX, CIP, E, CN, OFX	10	5
10	AMP, SXT, AMC, OX, CIP, E, CN, OFX, CTX	9	5

*AMP* ampicillin, *SXT* sulphomethoxazole–trimethoprim, *AMC* Amoxicillin–clavulanic acid, *OX* oxacillin, *CIP* ciprofloxacin, *E* erythromycin, *CN* gentamicin, *OFX* ofloxacin, *CTX* cefotaxime

Table 3 shows the minimum inhibitory concentration (MIC) distribution of the CoNS species. Based on the Clinical and Laboratory Standards Institute (CLSI) guidelines for MIC reading, the percentage of CoNS species resistant to the ampicillin, trimethoprim-sulfomethoxazole, amoxicillin-clavulanic acid and oxacillin were 85.5, 67.8, 49.8 and 25.9% respectively.

**Table 3:** Minimum inhibitory concentration (MIC) of antibiotics against CoNS isolated from fermented food samples

Antimicrobial agents	Numbers of CoNS with MIC^a^											Resistant isolates (%)
	≤0.12	0.25	0.5	1	2	4	8	16	32	64	128	
Ampicillin	24	13	*11*	*62*	*55*	*27*	*0*	*0*	*43*	*18*	*2*	218 (85.5%)
Trimethoprim–sulfomethoxazole	11	37	19	15	0	*0*	*0*	*0*	*0*	*70*	*103*	173 (67.8%)
Amoxicillin–clavulanic acid	101	0	27	0	0	0	*0*	*0*	*0*	*40*	*87*	127 (49.8%)
Oxacillin	53	136	0	*0*	*0*	*47*	*0*	*0*	*19*	*0*	*0*	66 (25.9%)
Ciprofloxacin	84	43	14	0	**52**	*0*	*7*	*24*	*9*	*9*	*13*	62 (24.3%)
Ofloxacin	21	12	22	69	**121**	*0*	*0*	*0*	*0*	*0*	*10*	10 (3.9%)
Gentamicin	15	151	16	**26**	**10**	**0**	*21*	*7*	*0*	*0*	*0*	37 (14.5%)
Erythromycin	98	91	15	0	0	0	*0*	*6*	*0*	*40*	*0*	46 (18.0%)
Cefotaxime^b^	0	181	20	0	0	49	0	0	0	3	2	–

^a^Based on the CLSI breakpoints. MICs indicative for susceptible isolates are displayed on a white background, those for intermediate on a bold and those for resistant on a italics

^b^Susceptility of staphylococci to cefotaxime may be detected from testing only penicillin and either cefoxitin or oxacillin

**Fig. 2:**
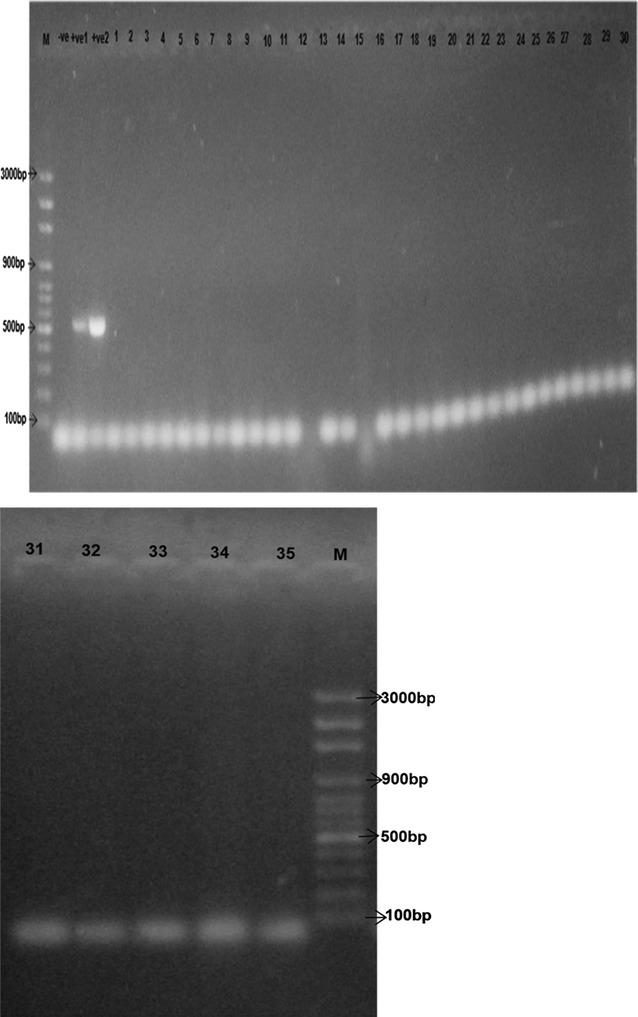
Gel electrophoresis micrograph of PCR product screen for *mecA* from extracted DNA (1–35) *Left* to *right*: M = O’GeneRuler 100 bp plus molecular weight marker (Thermo Scientific Fermentas™), Well 2 = Negative control for *mecA* gene, well 3 = Positive control 2, well 4 = Positive control 2 for *mecA* gene, wells 5–30 = Isolates 1–30. Weight of *mecA* gene = 513 bp. Positive isolates- none

Cefoxitin screening test was carried out on the isolates so as to establish their status as methicillin resistant strains. Thirteen (5.1%) of the tested isolates were positive to cefoxitin screening test and most of them were multidrug resistant with the highest occurrence of the methicillin resistant species in *nono*. The species were *S. kloosii* (KIL 4), *S. xylosus* (WAIL 3, KIL 2 and WAJ 5), *S. epidermidis* (OGIL 3, IRIL 7, NOMA 10, NOL 3 and IRIL 5), *S. simulans* (NOJ 6 and KIM 5) and *S. caprae* (NOMA 5 and NOMA 6) as indicated in Table 4. The molecular expression for *mecA* genes revealed that none of the isolates possessed the resistant genes (Fig. 2).

**Table 4:** Coagulase negative Staphylococci isolates showing methicillin resistance using cefoxitin screening

ID	Source	C–S	Resistance phenotype
*S. xylosus* KIL 2	*Kindirmo*	+	AMP, OX, SXT, AMC, CIP, E, CXT, CN. OFX
*S. xylosus* WAIL 3	*Wara*	+	AMP, OX, SXT, CIP, E, CXT, CN. OFX
*S. kloosii* KIL 4	*Kindirmo*	+	AMP, OX, SXT, AMC, CIP, E, CN OFX
*S. epidermidis* OGIL 3	*Ogi*	+	AMP, OX, AMC, CIP, E, OFX
*S. epidermidis* IRIL 7	*Iru*	+	AMP, OX, SXT, AMC, CIP, CN OFX
*S. epidermidis* NOMA 10	*Nono*	+	AMP, OX, SXT, AMC, CIP, E, CXT, CN OFX
*S. simulans* NOJ 6	*Nono*	+	AMP, OX, CIP, E, CXT, OFX
*S. simulans* KIM 5	*Kindirmo*	+	AMP, OX, SXT, AMC, CIP, E,
*S. xylosus* WAJ 5	*Wara*	+	AMP, OX, SXT, AMC, E, CXT
*S. caprae* NOMA 5	*Nono*	+	AMP, OX, SXT, AMC, CIP, E, CXT, OFX
*S. caprae* NOMA 6	*Nono*	+	AMP, OX, SXT, CIP, E, CN OFX
*S. epidermidis* NOL 3	*Nono*	+	AMP, OX, SXT, AMC, E, CXT CN OFX
*S. epidermidis* IRIL 5	*Iru*	+	AMP, OX, SXT, CIP, E, CN OFX

## Discussion

Majority of the CoNS strains exhibited resistance to ampicillin, amoxicillin-clavulanic acid, sulphomethoxazole–trimethoprim, and oxacillin. A large percentage of the strains were susceptible to the antibiotics ciprofloxacin, erythromycin, gentamicin, cefotaxime and ofloxacin. *Staphylococcus epidermidis* demonstrated the highest resistance to ampicillin while *S. xylosus* exhibited the highest resistance to sulfomethoxazole–trimethoprim. The highest percentage resistance to amoxicillin-clavulanic acid was demonstrated by *S. simulans*. Oxacillin resistance was highly demonstrated by *S. xylosus*. The resistance exhibited by a large percentage of CoNS to these routinely used antibiotics in treatment of staphylococcal infections necessitates the search for newer and more effective antibiotics against this group of organisms.

There was discrepancy between the detection of methicillin resistance phenotypically using cefoxitin screening and the absence of *mecA* gene in the CoNS strains. This may be attributed to methicillin resistance being caused by other mechanisms other than expression of *mecA* gene [24]. Also the sensitivity of PCR in the detection of *mecA* may have been compromised by the presence of PCR inhibitors or other physical factors [25, 26]. The work by [27] showed that out of 15 isolates showing oxacillin resistant phenotype, only one possessed the *mecA* gene, it was noted that there were unusual methicillin resistant CoNS that have a resistance mechanism other than the production of PBP2a and they have been reported as borderline methicillin resistant strains [28]. The borderline methicillin resistant strains are resistant to oxacillin due to their plasmid borne determinants including hyperproduced penicillinases, genes conferring resistance to cadmium or other gene products [29, 30]. It is also possible that the *mecA* negative oxacillin resistant CoNS may possess *mecA* alleles which could not be detected by the primers used in this study. Many CoNS strains also show diversity in *mecA* sequences and have different impact on β-lactam resistance.

## Conclusions

The high percentage of antimicrobial resistance demonstrated by the strains shows that food may serve as reservoirs for antibiotic resistance and allow for horizontal gene transfer from farm animals or their products to humans. Indigenous fermented food products may represent a critical risk for transfer of antimicrobial resistance to humans. As a consequence, transfer of antimicrobial resistance genes between bacteria after ingestion by humans may occur. Antimicrobial resistant CoNS present in food constitute a direct risk to public health as they increase the gene pool from which pathogenic bacteria can pick up resistance traits. The resistance of the organisms to routinely used antimicrobials also calls for the search for new antimicrobials and more effective management of diseases caused by CoNS in the event of an outbreak.

## Abbreviations

CoNS: coagulase-negative staphylococci
*mecA*: methicillin resistant gene
PBP2a: penicillin binding protein
DNA: deoxyribonucleic acid
CLSI: Clinical and Laboratory Standards Institute
MIC: minimum inhibitory concentration
